# MOMHCA-SG: a multi-head cross-attention and similar graph convolutional network framework for Alzheimer’s disease cell type classification

**DOI:** 10.3389/fnins.2026.1728558

**Published:** 2026-02-16

**Authors:** Yan Qian, Wei Kong, Shuaiqun Wang, Gen Wen, Yaling Yu

**Affiliations:** 1 College of Information Engineering, Shanghai Maritime University, Shanghai, China; 2 Department of Orthopedic Surgery, Shanghai Sixth People’s Hospital Affiliated to Shanghai Jiao Tong University School of Medicine, Shanghai, China; 3 Institute of Microsurgery on Extremities, Shanghai Sixth People’s Hospital Affiliated to Shanghai Jiao Tong University School of Medicine, Shanghai, China

**Keywords:** Alzheimer’s disease, cell classification, cell integration, deep learning, single-cell multi-omics data

## Abstract

**Introduction:**

Alzheimer’s disease (AD) is a complex neurodegenerative disorder with di-verse cellular and molecular characteristics. Due to its heterogeneous nature, early detection and understanding of AD are challenging, and conventional unimodal approaches often fail to capture the intricate interactions across different biological layers.

**Methods:**

We propose MOMHCA-SG, a novel multi-omics integration framework that combines a multi-head cross-attention mechanism (MHCA) with a graph convolutional network (GCN) guided by similarity network fusion (SNF). The framework first employs an autoencoder (AE) for dimensionality reduction and feature extraction, followed by MHCA to model inter-omic dependencies. A contrastive learning strategy is then utilized to refine latent representations, while the GCN performs final cell-type classification using fused similarity networks derived from both scRNA-seq and scATAC-seq datasets related to AD.

**Results:**

Extensive experiments on publicly available AD datasets demonstrate that MOMHCA-SG attains superior performance in both integration and classification tasks, achieving an ARI of 0.99, NMI of 0.98, and AMI of 0.98, with classification accuracy exceeding 0.98. Downstream bioinformatics analyses further identify key genes and signaling pathways potentially involved in AD pathogenesis, supporting the framework’s biological interpretability.

**Discussion:**

MOMHCA-SG effectively preserves feature integrity during high-dimensional processing, dynamically integrates heterogeneous omics data, and captures cross-modality relationships. These capabilities highlight its potential as a powerful tool for elucidating disease mechanisms and advancing precision medicine in neurodegenerative disorders.

## 1 Introduction

Alzheimer’s disease (AD) is a neurodegenerative disorder marked by neuronal loss and gliosis, leading to cognitive and memory deficits (Breijyeh and Karaman, 2020). Recent advances in single-cell multi-omics, particularly the integration of scRNA-seq and scATAC-seq, have enabled multidimensional insights into AD mechanisms (Hwang et al., 2018; Grandi et al., 2022). This combined analysis reveals cell-type-specific regulatory networks (Zhang and Zhang, 2019) (Stuart and Satija, 2019) (Dou et al., 2022), improving both transcriptional regulation inference and cell-type annotation accuracy (Rahimzadeh et al., 2024). While scRNA-seq classifies cells based on marker gene expression, scATAC-seq identifies subtypes through promoter accessibility (Cui et al., 2024). Their integration corrects single-omics bias and enables the detection of rare subpopulations, enhancing classification robustness. However, technical differences and data heterogeneity between omics still pose challenges to integrated analysis, limiting the improvement of existing methods in classification accuracy and generalization ability.

Numerous integration strategies have been proposed to address the challenges of single-cell multi-omics analysis. Traditional approaches like Seurat (Hao et al., 2021) and scAI (Jin et al., 2020), based on generalized linear models, struggle with capturing the complex nonlinearities in single-cell data. MOFA+ (Argelaguet et al., 2020) employs variational inference for low-dimensional modeling across multiple data modalities, while Mowgli (Huizing et al., 2023) leverages non-negative matrix factorization with optimal transport to improve clustering interpretability. Other methods, such as SCIM (Stark et al., 2020) and LIGER (Liu et al., 2020), perform well under low-complexity scenarios but lack scalability to intricate inter-omic relationships. With the rise of deep learning, more sophisticated models have emerged. DSIR (Yang et al., 2022) extracts global and local structures via deep integration strategies, and DCCA (Zuo et al., 2021) aligns omics using variational AEs and attention transfer. scMM (Minoura et al., 2021) introduces multimodal variational AEs to enable interpretable representation learning and cross-modal generation. scMCs (Ren et al., 2023) combines auto-encoders, multi-head attention, and contrastive learning to jointly model shared and specific features, enhancing clustering and imputation. SSGATE (Lv et al., 2024) applies a dual-path graph attention AE for self-supervised integration of single-cell and spatially re-solved data. Despite these advances, most models still face difficulties in resolving inter-omic heterogeneity, often resulting in poor intra-cluster cohesion and insufficient separation of distinct cell types.

In cell type classification, few models fully exploit the potential of single-cell multi-omics data. Many existing methods still focus on single-omics. For instance, scPred (Alquicira-Hernandez et al., 2019) applies feature selection and probabilistic classification within reduced-dimensional scRNA-seq data, while scClassify (Lin et al., 2020) uses ensemble learning for multi-scale classification within the same modality. Some methods have extended to multi-omics integration. MoGCN (Li et al., 2022) combines GCNs, AEs, and similarity net-works for cancer subtype classification. MOGAT (Tanvir et al., 2024) applies graph attention mechanisms across multiple omics for the same task. scMoMtF (Lan et al., 2024) integrates multimodal embeddings through fully connected networks to enhance cell type classification. Despite these advances, current methods still face difficulties in modeling complex inter-omic interactions and improving generalizability across diverse datasets.

To address the shortcomings of existing methods in multi-omics data integration and cell type classification, this study proposed a MHCA and similarity GCN framework (MOMHCA-SG) for cell type classification. Specifically, this study first applies an AE to perform dimensionality reduction on multi-omics expression data and extract key features. Next, the study employs a MHCA to effectively overcome the limitations of traditional methods in capturing the complex interactions between omics data. By adaptively learning the relationships between different omics layers, MHCA enhances the accuracy of data integration. Furthermore, contrastive learning is introduced to optimize latent space representations, ensuring that similar samples are clustered together in the latent space while dissimilar samples are separated, thereby improving clustering performance. Subsequently, a SNF method is employed to integrate network topological information from multi-omics data, constructing a similarity network of cells and providing a comprehensive view of the cells. Finally, the integration results from MHCA and SNF are combined, and a GCN is used to build the final model for cell type classification. Unlike some methods that rely on explicit distributional assumptions, the MOMHCA-SG framework does not impose any predefined distribution on the underlying features. Both the core AE and MHCA modules do not require the data to follow a Gaussian distribution. Consequently, the framework remains applicable even when real-world biological data deviate from idealized distributions. Experimental results demonstrate that, compared to current state-of-the-art methods, the dimensionality-reduced data from MOMHCA-SG better distinguishes cell subtypes and exhibits higher clustering consistency. In the task of multi-omics data integration, MOMHCA-SG was applied alongside advanced integration methods such as Mowgli, scMDC, and SSGATE to an AD-related single-cell multi-omics dataset, which included scRNA-seq and scATAC-seq data. MOMHCA-SG showed superior performance in data integration. In the cell classification task, a further comparison was made between MOMHCA-SG and classification methods such as MoGCN, scMoMtF, and MOGAT. Ten-fold cross-validation results revealed that MOMHCA-SG achieved significantly higher accuracy in cell classification compared to the other methods. Overall, the comprehensive experimental findings confirm that MOMHCA-SG excels in both single-cell data integration and classification tasks.

## 2 Materials and methods

### 2.1 Datasets

To evaluate the model’s performance, real-world single-cell multi-omics datasets were obtained from multiple platforms. These include GSE214979^1^ and GSE140203^2^ from the GEO database, and the 10x Genomics Multiome-PBMC 10k dataset. The datasets encompass various species, including humans and mice, and include corresponding scRNA-seq and scATAC-seq profiles.

The GSE214979 dataset specifically includes multi-omics data extracted from the cortical tissues of seven AD patients and eight unaffected individuals. It provides rich information on gene expression and cell type-specific transposase-accessible regions, useful for identifying potential cis-regulatory elements (CREs) linked to transcriptional alterations in AD. GSE140203 involves SHARE-seq-based profiling of mouse lung, skin, and brain tissue, offering joint measurements of chromatin accessibility and gene expression. This dataset enables fine-grained regulatory mapping across cell types and reveals both cis- and trans-regulatory programs.

Table 1 summarizes dataset characteristics, including cell counts, gene numbers in scRNA-seq, peak counts in scATAC-seq, cell types, and species.

**Table 1:** Detailed information of single-cell multi-omics datasets.

Dataset	Cells	Genes	Peaks	Cell types	Species
GSE214979	5,164	36,601	150,614	8	*Homo sapiens*
PBMC-10k	9,631	29,095	107,194	19	*Homo sapiens*
GSE140203	5,692	21,478	340,341	22	*Mus musculus*

### 2.2 Data preprocessing

In this study, preprocessing of scRNA-seq and scATAC-seq data was conducted in R (v4.4.1) using the Seurat and Signac frameworks. Given the substantial differences between transcriptomic and chromatin accessibility data in terms of scale, distributional properties, and sparsity, modality-specific preprocessing strategies were implemented prior to multi-omics integration.

For scRNA-seq data, quality control (QC) was first performed based on the number of detected genes per cell and the proportion of mitochondrial gene expression to exclude low-quality cells and potential doublets. Cells with 300–10,000 detected genes and <5% mitochondrial reads were retained. Genes not expressed in any cell were removed to reduce background noise. The expression matrix was then normalized and log-transformed using the global LogNormalize method (scale factor = 10,000). Highly variable genes (HVGs) were identified using the variance-stabilizing transformation (VST) approach for downstream analyses, enabling retention of major biological variation while mitigating technical noise. Principal component analysis (PCA) was subsequently applied to the selected HVGs for dimensionality reduction. Finally, the selected features were centered and scaled to reduce the influence of extreme expression values.

For scATAC-seq data, stringent filtering was performed using chromatin accessibility-specific QC metrics, including transcription start site (TSS) enrichment score, nucleosome signal, the number of fragments in peak regions, and the fraction of fragments overlapping blacklist regions. High-quality cells were retained if they contained 2,000–20,000 peak fragments, exhibited a blacklist fraction <5%, had a nucleosome signal <4, and showed a TSS enrichment score >3. Considering the high sparsity and near-binary nature of scATAC-seq profiles, term frequency-inverse document frequency (TF-IDF) normalization was applied, followed by selection of the most informative peaks (top peaks) using the FindTopFeatures function. Latent semantic indexing (LSI) was then performed on the selected peaks for dimensionality reduction, capturing major sources of variation while alleviating the intrinsic sparsity of scATAC-seq data. Finally, features were scaled to attenuate the impact of outlier signals.

### 2.3 Overview of MOMHCA-SG

**Figure 1:**
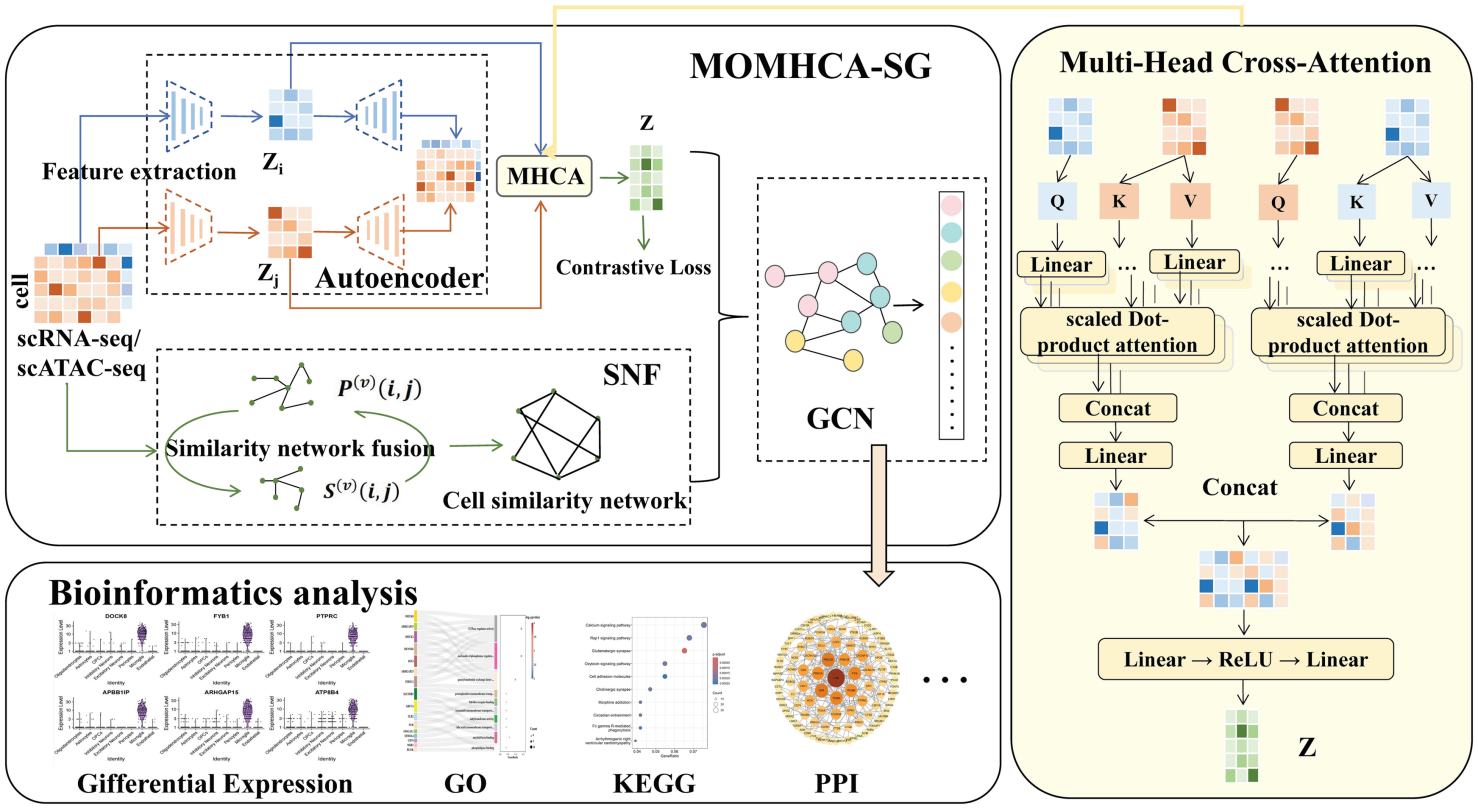
Workflow of this study.

MOMHCA-SG comprises three key components: the MOMHCA-based integration module, the SNF-based network topology module, and the GCN-based classification module. The architecture of MOMHCA-SG is illustrated in Figure 1.

This model takes single-cell multi-omics data, specifically scRNA-seq and scATAC-seq, as input. The process involves three main stages: (1) Feature extraction and data integration: Initially, AE are employed to extract features from each omics dataset. Subsequently, an integration module based on the MHCA mechanism is introduced. This module adaptively weights and combines features from various omics layers, producing a consolidated latent representation. The representation is then optimized through a contrastive learning strategy, which enhances clustering by maximizing the aggregation of similar cells while improving the distinction between different cell clusters, ensuring that the integrated latent space is more conducive to clustering. (2) SNF: In the second step, the features derived from different omics data are integrated using the SNF method to construct a cell similarity network. (3) Cell Type Classification: Finally, the fused features and cell similarity network are combined, and a GCN is employed for cell type classification, enabling efficient and accurate identification of cell types.

### 2.4 Multi-omics integration module based on multi-head cross-attention mechanism

Single-cell datasets are inherently high-dimensional, capturing diverse biological signals. To uncover intercellular relationships and compress these complex features, this study adopts an AE framework that encodes cell-level information into a latent space while preserving structural integrity. The AE architecture is composed of an encoder that projects the input *X* into a lower-dimensional latent vector *Z*, and a decoder that recon-structs the input from *Z*, producing *X*′. These transformations are formally defined as: *z* = *f*(*W*(1)*x* + *b*(1)) and *x*′ = *g*(*W*(2)*z* + *b*(2)). The AE is trained to minimize reconstruction discrepancy using the mean squared error (MSE) as the loss function: *L* = MSE (*x*, *x*′). This allows the model to preserve essential biological features throughout the compression and reconstruction processes.

The study integrates two omics modalities: scRNA-seq and scATAC-seq. The scRNA-seq data are represented as a matrix $X_{i}\in {\mathbb{R}}^{N\times D_{1}}$ , where *N* denotes the number of cells and $D_{1}$ corresponds to the number of gene expression features. In contrast, scATAC-seq data are organized into a matrix $X_{j}\in {\mathbb{R}}^{N\times D_{2}}$ , with $D_{2}$ indicating the number of chromatin accessibility peaks.

To process both data types, the model employs two distinct encoder-decoder pathways—one per omics modality—which converge into a shared latent representation space. This unified space facilitates multi-omics integration while allowing each encoder-decoder branch to learn domain-specific transformations. The overall training objective incorporates reconstruction errors from both modalities and is expressed as Equation 1.


$$L=\alpha \cdot \mathrm{MSE}(X_{i},X_{i}^{\prime })+\beta \cdot \mathrm{MSE}(X_{j},X_{j}^{\prime })$$ (1)

here, $X_{i}$ and $X_{j}$ represent the original input data, while $X_{i}^{\prime }$ and $X_{j}^{\prime }$ are the data reconstructed by the decoders. The weights of each omics data type must satisfy α+β=1 .

After passing through several transformation layers, the model generates compact embedding representations $Z_{i}\in {\mathbb{R}}^{N\times d}$ and $Z_{j}\in {\mathbb{R}}^{N\times d}$ for each omics modality. These embeddings capture the structural and feature characteristics of scRNA-seq and scATAC-seq data. However, naive strategies like direct concatenation or averaging can obscure essential information or introduce irrelevant signals due to modality-specific heterogeneity.

scRNA-seq and scATAC-seq reside in distinct feature spaces and exhibit substantial differences in sparsity and noise characteristics. To effectively model cross-omics relationships, we employ a MHCA for dynamic integration. Unlike self-attention (Vaswani et al., 2017), which focuses on interactions within a single modality, cross-attention (Gheini et al., 2021) explicitly models conditional interactions between different omics by treating one modality as queries and the other as keys and values. This design enables the model to exploit complementary information across modalities to derive a shared latent representation *Z*. Attention matrices are learned, and linear transformations are applied to generate feature representations for both data types. For scRNA-seq, the query, key, and value representations are computed as: $Q_{i}=Z_{i}W^{Q}$ , $K_{i}=Z_{j}W^{K}$ , $V_{i}=Z_{j}W^{V}$ . Similarly, for scATAC-seq: $Q_{j}=Z_{j}W^{Q}$ , $K_{j}=Z_{i}W^{K}$ , $V_{j}=Z_{i}W^{V}$ , where $W^{Q}\in {\mathbb{R}}^{d\times d_{k}}$ , $W^{K}\in {\mathbb{R}}^{d\times d_{k}}$ , $W^{V}\in {\mathbb{R}}^{d\times d_{k}}$ are learnable projection matrices.

Attention scores are obtained by computing scaled dot products between queries and keys, as shown in Equation 2.


$$\text{Attention}(Q,K,V)=\text{softmax}(\frac{Q\cdot K^{⊤}}{\sqrt{d_{k}}})\cdot V$$ (2)

This operation measures similarity between modalities within the latent space. The Softmax function ensures numerical stability by transforming scores into probabilities. Using this mechanism, each attention head generates output representations. For scRNA-seq and scATAC-seq, head *h* is computed as shown in Equations 3, 4.


$${\text{head}}_{\mathrm{ih}}=\text{Attention}(Z_{i}W_{h}^{Q},Z_{j}W_{h}^{K},Z_{j}W_{h}^{V})$$ (3)

$${\text{head}}_{\mathrm{jh}}=\text{Attention}(Z_{j}W_{h}^{Q},Z_{i}W_{h}^{K},Z_{i}W_{h}^{V})$$ (4)

All heads are concatenated into a unified vector and projected via a weight matrix *W* to complete the integration, as shown in Equation 5.


$$\text{head}=\text{MultiHead}(Q,K,V)=\text{concat}({\text{head}}_{1},\ldots ,{\text{head}}_{h})\cdot W$$ (5)

here, *h* denotes the number of attention heads, and the final output has dimensionality *d*′ = *h* ⋅ *d_k_*, mapped to the target space through $W\in {\mathbb{R}}^{d^{\prime }\times d}$ .

This structure enables the model to capture complex inter-modal dependencies within the latent space, promoting effective alignment and fusion. To enhance training stability and integration consistency, a residual connection is added, followed by layer normalization: head′ = LayerNorm (head + *Z*). The final refined embeddings are denoted as ${\text{head}}_{i}^{\prime }$ and ${\text{head}}_{j}^{\prime }$ for the respective omics data.

Next, this study introduces a fusion module, which further integrates the cross-attention of both groups to generate a unified latent representation *Z*. This module concatenates ${\text{head}}_{i}^{\prime }$ and ${\text{head}}_{j}^{\prime }$ , followed by a non-linear transformation through a feed-forward neural network, thereby enhancing the cross-group interaction and improving the joint representation capacity. The architecture is expressed as follows in Equation 6.


$$Z=\mathrm{FFN}([{\text{head}}_{i}^{\prime }{\text{head}}_{j}^{\prime }])$$ (6)

here, ∥ denotes concatenation; the FFN refers to a feed-forward network consisting of two layers, coupled with a non-linear activation function. The feed-forward network is formulated as shown in Equation 7.


$$\mathrm{FFN}(x)=\sigma (xW_{1}+b_{1})W_{2}+b_{2}$$ (7)

Finally, the unified representation *Z* serves as the input to the subsequent layers of the network.

### 2.5 Comparative learning

To integrate single-cell multi-omics data from heterogeneous sources with varying expression dimensions, this study introduced an omics fusion module based on a MHCA. This module calculates attention weights between two omics groups and combines the features accordingly, effectively producing a cohesive latent representation. This approach mitigates the interference of omic heterogeneity on integration consistency and accuracy. However, the feature embeddings obtained solely through the attention mechanism may not fully ensure the intra-class compactness (e.g., within the same cell type or state) in the latent space, nor the inter-class discriminability between omics groups, thereby limiting the generalization capacity of the representation learning.

To address this limitation, this study further introduces a supervised contrastive learning strategy by constructing a contrastive loss function, which explicitly leverages available cell-type annotations during training, and pulls together samples from the same class in the latent space while pushing apart samples from different classes, thereby enhancing the discriminative power. Let a point $a\in {\mathbb{R}}^{N\times d}\;$ represent the feature vector in the latent space, and define the positive sample $a^{+}$ as one drawn from the same cell type as the anchor cell, along with a set of negative samples $\left\{a_{1}^{-},a_{2}^{-},\ldots ,a_{n}^{-}\right\}$ drawn from different cell types. The contrastive loss function is defined in Equation 8.


$$L=-\log\frac{\exp(s(a,a^{+})/\tau )}{\exp(s(a,a^{+})/\tau )+\sum_{j=1}^{N-1}\exp(s(a,a^{-})/\tau )}$$ (8)

where τ denotes the temperature parameter that controls the distribution of similarities; *s* (·,·) represents the similarity function. This optimization process significantly enhances the latent representations’ ability to maintain class compactness and separability. The module further strengthens the organization of latent space, laying the foundation for improved performance in downstream tasks such as clustering, classification, and biological marker recognition.

### 2.6 SNF-based graph convolutional network classification framework

To fully harness the complementary information between scRNA-seq and scATAC-seq data, this study employs a SNF method to construct a cellular similarity graph structure. The GCN is then utilized for cell type classification.

Let *n* represent the number of cell samples and *v* the number of omics data types. First, compute the Euclidean distance between cells in each omic space: $\rho^{\left(v\right)}(i,j)=\|X_{i}^{\left(v\right)}-{X_{j}^{\left(v\right)}}_{2}\|$ . For each omic data type, the cell-to-cell similarity matrix is calculated. The similarity matrix for the *v*-th omic data type is defined as: $W^{\left(v\right)}\in {\mathbb{R}}^{n\times n}$ , with the elements of the matrix defined as shown in Equation 9.


$$W^{\left(v\right)}(i,j)=\exp(-\frac{\rho^{\left(v\right)}{\left(i,j\right)}^{2}}{\mu \cdot \varepsilon_{\mathrm{ij}}})$$ (9)

where $x_{i}^{\left(v\right)}\;$ and $x_{j}^{\left(v\right)}$ represent the feature vectors of the *i*-th and *j*-th cells in the *v*-th omic data type, respectively; ρ (·,·) represents the Euclidean distance between cells within the feature space; $\varepsilon_{\mathrm{ij}}$ is a predefined hyperparameter controlling the scale of the distance metric.

To enhance local structure information, this study further defines the cell-to-cell similarity matrix $P^{\left(v\right)}$ and the K-nearest neighbor similarity matrix $S^{\left(v\right)}$ under omic data type *v*, as defined in Equations 10, 11, where:


$$P^{\left(v\right)}(i,j)=\{\begin{array}{cc} \frac{W^{\left(v\right)}(i,j)}{2\sum_{k\neq i}W^{\left(v\right)}(i,k)}, & j\neq i\\ \frac{1}{2}, & j=i \end{array}$$ (10)

$$S^{\left(v\right)}(i,j)=\{\begin{array}{cc} \frac{W^{\left(v\right)}(i,j)}{\sum_{k\in N_{i}}W^{\left(v\right)}(i,k)}, & j\in N_{i}\\ 0, & \text{otherwise} \end{array}$$ (11)

where $N_{i}$ represents the set of *K*-nearest neighbors for the *i*-th cell. Initially, at *t* = 0, set $P_{0}^{\left(1\right)}=P^{\left(1\right)}$ , $P_{0}^{\left(2\right)}=P^{\left(2\right)}$ , and then iteratively update the similarity matrices via a message-passing mechanism to propagate structural information between the omics: $P_{t+1}^{\left(1\right)}=S^{\left(1\right)}\cdot P_{t}^{\left(2\right)}\cdot {\left(S(1)\right)}^{T}$ , $P_{t+1}^{\left(3\right)}=S^{\left(2\right)}\cdot P_{t}^{\left(1\right)}\cdot {\left(S^{\left(2\right)}\right)}^{T}$ (Li et al., 2022). After *t* iterations, the final fused similarity matrix $P\in {\mathbb{R}}^{n\times n}$ is obtained by averaging the two omic-based similarity matrices, as shown in Equation 12.


$$P=\frac{P_{t}^{\left(1\right)}+P_{t}^{\left(2\right)}}{2}$$ (12)

The fused matrix $P\in {\mathbb{R}}^{n\times n}$ represents the integrated similarity structure of cells across multiple omics datasets, serving as the adjacency matrix *A* for the GCN classification model. Simultaneously, based on multi-head attention mechanisms, the multi-omics fusion module outputs $Z\in {\mathbb{R}}^{n\times d}$ , which is defined as the feature matrix *H* of the graph nodes. Each layer of the GCN updates the node representations through graph convolution, as described in Equation 13.


$$H^{\left(l\right)}=\sigma (\tilde{A}\cdot H^{\left(l-1\right)}\cdot W^{\left(l\right)})$$ (13)

where $W^{\left(l\right)}$ represents the learnable weight matrix at layer *l*, and σ(⋅) is the activation function. The matrix $\tilde{A}=D^{-(1/2)}AD^{-(1/2)}$ is the normalized adjacency matrix, with *D* being the degree matrix and *A* the adjacency matrix. This multi-layer network enables the propagation of cell similarity information, capturing both local and global feature representations. The final output layer produces the fully connected graph that predicts the class label for each cell.

### 2.7 Comparison of methods and evaluation metrics

To assess the integration performance of MOMHCA-SG, we carried out a comprehensive comparison with six leading multi-omics integration approaches: Mowgli (Huizing et al., 2023), DCCA (Zuo et al., 2021), scMM (Minoura et al., 2021), scMCs (Ren et al., 2023), SSGATE (Lv et al., 2024), and scMDC (Lin et al., 2022). All methods were paired with the k-means clustering algorithm (Ahmed et al., 2020), and hyperparameters were set according to their original publications. Performance was assessed using three standard clustering metrics: Adjusted Rand Index (ARI) (Steinley, 2004), Normalized Mutual Information (NMI) (Knops et al., 2006), and Adjusted Mutual Information (AMI) (Vinh et al., 2009). For classification, MOMHCA-SG was benchmarked against three leading multi-omics classifiers: MoGCN (Li et al., 2022), MOGAT (Tanvir et al., 2024), and scMoMtF (Lan et al., 2024), using the original parameter configurations. Evaluation metrics included accuracy (ACC) (Cai et al., 2005), weighted *F*1-score (Chicco and Jurman, 2020), and precision (Buckland and Gey, 1994).

In the integration task, all comparison methods, including scMM, DCCA, scMDC, Mowgli, SSGATE, and scMCs, were implemented using their publicly available code. For each comparison method, the model architecture and hyper-parameters were set according to recommendations in the corresponding original literature, using default settings or best-performing configurations reported in the literature. No additional hyper-parameter tuning was performed to favor or weaken any particular method. To ensure fair comparison, all methods were applied to the same preprocessed dataset. For methods involving randomized optimization or randomized initialization, experiments were repeated multiple times using different random seeds, and performance metrics were reported as the average of multiple runs. Although the primary design goals of the aforementioned methods differ, they all aim to learn a joint low-dimensional representation of single-cell multi-omics data, which can serve as the basis for various downstream analytical tasks. To compare the quality of the joint representation learned by different methods within a unified and fair evaluation framework, this study uses the same k-means clustering algorithm to cluster cells based on the integrated representation output by each method, thereby avoiding potential biases due to different clustering strategies and focusing the comparison results more on the performance differences in representation learning itself.

### 2.8 Implementation details and hyperparameter settings

For each omics dataset, we use an AE to extract features. Each encoder consists of two fully connected layers with dimensions $d_{m}$ → 512 → 128, where $d_{m}$ ​represents the input feature dimension of the corresponding omics dataset. Batch normalization and the GELU activation function are applied after the intermediate layers. The decoder is implemented using linear layers to map the latent space back to the original feature space. The latent dimension is fixed at 128 in all experiments.

To integrate heterogeneous omics representations, we introduce a MHCA module with four attention heads. Given two latent representations, we apply cross-attention bidirectionally to capture inter-omics dependencies. The attention outputs are fused through residual connections and layer normalization. Subsequently, we use a fusion network consisting of two fully connected layers (with ReLU activation function) to merge the attention-focused representations into a unified latent embedding.

To enhance the discriminative power between categories in the latent space, supervised contrastive learning was applied during training. Latent embeddings were L2 normalized, and pairwise cosine similarity was calculated. Positive pairs consisted of cells with the same cell type label, while negative pairs corresponded to cells of different types. A temperature parameter *τ* = 0.5 was used to control the sharpness of the similarity distribution. Contrastive loss was jointly optimized with the reconstruction objective.

Subsequently, using the SNF framework, we constructed inter-cell similarity networks for each type of omics data. The affinity matrix was calculated using the squared Euclidean distance metric, with a nearest neighbor count of 9 and a normalization parameter of *φ* = 0.5. The various affinity networks were iteratively fused to obtain a unified cell similarity network, which was then used as the adjacency matrix for graph-based learning.

Finally, for cell type classification, we employed a two-layer GCN. This GCN consists of two graph convolutional layers using the ELU activation function, followed by a dropout regularization layer and a fully connected output layer. The hidden layer dimension was set to 64, and the output layer dimension corresponded to the number of cell types. We optimized the model using the Adam optimizer with a learning rate of 0.001 and a weight decay of 0.01. The training epochs were 150. Supervision information (labels) was limited to the training set; the test set labels were not used during training, and performance evaluation was based entirely on the reserved test set.

All hyper-parameters were selected based on previous research and validated using only the training data. Hyper-parameter tuning was performed on the training set of the cross-validation framework; the test set was not used for model selection or parameter tuning. Once all parameters were determined, they were uniformly applied to all datasets, cross-validation folds, and random seeds to ensure fair comparisons and reproducible results.

## 3 Experimental study

### 3.1 Single-cell multi-omics data integration performance

Tables 2–4 summarize the performance of MOMHCA-SG versus six state-of-the-art multi-omics integration methods across three single-cell datasets. The evaluation was conducted using *k*-means clustering, with results averaged over 5 independent runs under a 5-fold stratified cross-validation scheme. Specifically, the entire dataset was partitioned into five equal folds; in each round, one fold was used as the test set while the remaining four served as the training set, ensuring that every fold was used exactly once for testing. Importantly, stratification was applied so that the label distribution within each fold closely matched that of the overall dataset, thereby preventing class imbalance in individual folds and providing a more reliable and unbiased assessment of model performance.

**Table 2:** Performance of various methods on the GSE214979 dataset.

Method	ARI (%)	NMI (%)	AMI (%)
scMM	57.78 (56.85–58.77)	82.12 (80.39–84.77)	81.83 (80.08–84.52)
DCCA	68.36 (58.80–76.76)	71.95 (60.86–81.35)	80.73 (71.21–87.96)
scMDC	87.65 (87.15–88.96)	87.69 (87.37–87.90)	73.71 (85.33–87.85)
Mowgli	58.38 (34.59–97.95)	81.25 (69.63–95.49)	81.17 (69.34–95.47)
SSGATE	51.15 (41.28–74.86)	79.61 (74.02–85.43)	79.55 (73.94–85.39)
scMCs	51.34 (45.15–60.26)	67.93 (66.86–69.21)	75.64 (71.48–78.56)
**MOMHCA-SG**	**99.08 (98.34–99.36)**	**98.12 (97.06–99.72)**	**98.05 (96.90–98.70)**

**Table 3:** Performance of various methods on the PBMC-10k dataset.

Method	ARI (%)	NMI (%)	AMI (%)
scMM	32.16 (31.89–34.54)	62.65 (62.58–63.27)	61.05 (60.85–62.83)
DCCA	52.30 (46.70–54.80)	75.80 (74.50–76.80)	75.60 (74.30–76.70)
scMDC	32.50 (24.92–41.40)	59.85 (54.82–65.90)	59.57 (53.84–65.65)
Mowgli	36.29 (34.51–37.88)	69.93 (68.69–70.94)	69.64 (68.39–70.67)
SSGATE	56.05 (50.23–61.20)	77.15 (75.32–78.86)	76.99 (75.15–78.71)
scMCs	38.10 (34.50–41.90)	67.30 (65.20–69.90)	67.10 (64.90–69.70)
**MOMHCA-SG**	**91.05 (88.37–92.70)**	**89.48 (88.74–90.52)**	**89.09 (88.33–90.17)**

**Table 4:** Performance of various methods on the GSE140203 dataset.

Method	ARI (%)	NMI (%)	AMI (%)
scMM	17.36 (17.36–17.36)	41.81 (41.81–41.81)	37.44 (37.44–37.44)
DCCA	50.60 (46.00–53.90)	71.00 (68.20–72.70)	70.60 (67.70–72.30)
scMDC	22.52 (17.57–32.69)	42.02 (39.66–45.54)	41.15 (38.76–44.73)
Mowgli	28.13 (24.06–34.39)	46.98 (40.69–56.19)	46.09 (39.71–55.43)
SSGATE	56.11 (53.17–58.43)	71.69 (69.84–73.01)	71.28 (69.40–72.62)
scMCs	39.60 (36.70–43.10)	62.60 (61.00–64.70)	62.10 (60.50–64.20)
**MOMHCA-SG**	**70.03 (67.00–72.39)**	**79.80 (78.46–81.04)**	**78.23 (77.52–79.57)**

### 3.2 Classification performance of single-cell multi-omics data

**Figure 2:**
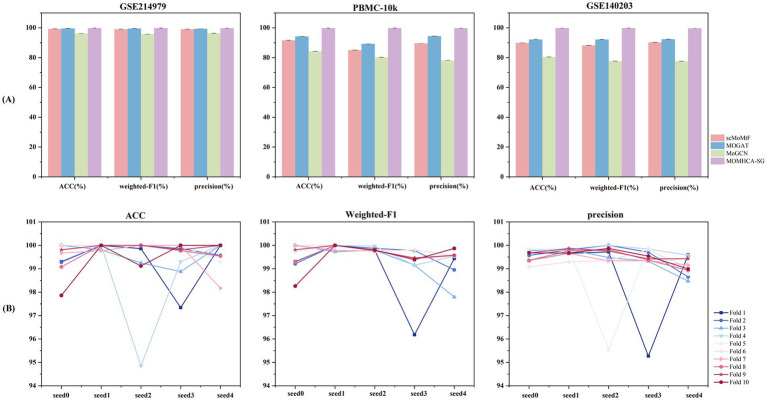
Comparative analysis of model performance with cross-validation. **(A)** Comparison of classification performance of MOMHCA-SG and other algorithms on multiple single-cell multi-omics datasets. **(B)** 10-fold cross-validation performance on GSE214979 dataset.

Figure 2A Compares MOMHCA-SG with three classification algorithms across three single-cell multi-omics datasets. We evaluated the MOMHCA-SG model using a 10-fold cross-validation scheme with group-wise splitting to avoid information leakage in multi-donor data. Specifically, we performed donor-level partitioning, such that all cells originating from the same donor were assigned exclusively to either the training set or the test set within each fold. In each iteration, the model was trained on cells from nine folds and evaluated on the remaining fold, ensuring that the test set contained donors unseen during training. To further account for variability in model initialization and training, the experiments were repeated across five different random seeds, and the results were averaged across these runs. MOMHCA-SG consistently achieved over 95% accuracy on all datasets, demonstrating strong robustness and generalizability. For the PBMC-10k dataset, it attained Weighted-*F*1: 99.43%, ACC: 99.37%, and Precision: 99.33%, surpassing the second-best method by 9.63, 5.17, and 5.13%, respectively.

As illustrated in Figure 2B, the performance variability across different cross-validation folds on the GSE214979 dataset is further examined under multiple random seeds. For example, under seed 0, MOMHCA-SG achieved a mean ACC of 99.476% across folds with a standard deviation of 0.66, together with a mean weighted *F*1-score of 99.558% (±0.56) and a mean precision of 99.547% (±0.25), indicating highly stable performance. In contrast, under seed 2, the model obtained a mean ACC of 99.308% with a larger fold-wise variation (±1.60), while the corresponding mean *F*1-score and precision were 99.802% (±0.07) and 99.290% (±1.34), respectively. These results demonstrate that MOMHCA-SG maintains consistently high performance across different random initializations while exhibiting reasonable variability across folds under donor-level cross-validation.

As shown in Tables 5–7, to assess the statistical significance of the observed performance improvements, paired t-tests and Wilcoxon signed-rank tests were conducted between MOMHCA-SG and the three baseline classification algorithms. The evaluation was based on 100 paired observations, derived from the test sets of a 10-fold cross-validation repeated across five different random seeds, ensuring a robust estimation of model performance. The results indicate that MOMHCA-SG significantly outperformed all baseline methods in terms of ACC, Weighted-*F*1, and precision.

**Table 5:** Statistical comparison on the GSE214979 dataset.

Metric	Method 1	Method 2	*t*-statistic	*p*-value (*t*-test)	*W* (Wilcoxon)	*p*-value (Wilcoxon)
ACC	MOMHCA-SG	MoGCN	27.43	4.54*E* − 48	1	4.01*E* − 18
	MOMHCA-SG	MOGAT	6.89	5.01*E* − 10	843	7.32*E* − 09
	MOMHCA-SG	scMoMtF	13.05	3.05*E* − 23	176	6.65*E* − 16
Weighted-*F*1	MOMHCA-SG	MoGCN	41.39	2.56*E* − 64	0	3.89*E* − 18
	MOMHCA-SG	MOGAT	8.12	1.28*E* − 12	647	1.06*E* − 10
	MOMHCA-SG	scMoMtF	7.42	4.06*E* − 11	715	4.86*E* − 10
Precision	MOMHCA-SG	MoGCN	52.43	4.88*E* − 74	0	3.89*E* − 18
	MOMHCA-SG	MOGAT	11.87	9.39*E* − 21	274	9.96*E* − 15
	MOMHCA-SG	scMoMtF	10.11	6.15*E* − 17	399	2.67*E* − 13

**Table 6:** Statistical comparison on the PBMC-10k dataset.

Metric	Method 1	Method 2	*t*-statistic	*p*-value (*t*-test)	*W* (Wilcoxon)	*p*-value (Wilcoxon)
ACC	MOMHCA-SG	MoGCN	915.51	3.01*E* − 196	0	3.89*E* − 18
	MOMHCA-SG	MOGAT	1345.98	8.11*E* − 213	0	3.89*E* − 18
	MOMHCA-SG	scMoMtF	284.39	5.27*E* − 146	0	3.89*E* − 18
Weighted-*F*1	MOMHCA-SG	MoGCN	1301.98	2.18*E* − 211	0	3.89*E* − 18
	MOMHCA-SG	MOGAT	2613.42	2.41*E* − 241	0	3.89*E* − 18
	MOMHCA-SG	scMoMtF	477.979	2.60*E* − 168	0	3.89*E* − 18
Precision	MOMHCA-SG	MoGCN	1387.320	4.07*E* − 214	0	3.89*E* − 18
	MOMHCA-SG	MOGAT	1141.20	1.01*E* − 205	0	3.89*E* − 18
	MOMHCA-SG	scMoMtF	363.83	1.38*E* − 156	0	3.89*E* − 18

**Table 7:** Statistical comparison on the GSE140203 dataset.

Metric	Method 1	Method 2	*t*-statistic	*p*-value (*t*-test)	*W* (Wilcoxon)	*p*-value (Wilcoxon)
ACC	MOMHCA-SG	MoGCN	1437.97	1.17*E* − 215	0	3.89*E* − 18
	MOMHCA-SG	MOGAT	1751.93	3.78*E* − 224	0	3.89*E* − 18
	MOMHCA-SG	scMoMtF	360.27	3.66*E* − 156	0	3.89*E* − 18
Weighted-*F*1	MOMHCA-SG	MoGCN	1392.44	2.83*E* − 214	0	3.89*E* − 18
	MOMHCA-SG	MOGAT	1496.97	2.18*E* − 217	0	3.89*E* − 18
	MOMHCA-SG	scMoMtF	323.22	1.68*E* − 151	0	3.89*E* − 18
Precision	MOMHCA-SG	MoGCN	1392.44	2.83*E* − 214	0	3.89*E* − 18
	MOMHCA-SG	MOGAT	1496.97	2.18*E* − 217	0	3.89*E* − 18
	MOMHCA-SG	scMoMtF	323.22	1.68*E* − 151	0	3.89*E* − 18

Overall, these findings demonstrate that MOMHCA-SG not only achieves superior average performance but also exhibits high consistency at the sample level and strong statistical robustness, further highlighting its effectiveness in handling diverse single-cell multi-omics datasets and varying experimental conditions.

### 3.3 Hyperparameter analysis

The performance of MOMHCA-SG is sensitive to hyperparameter settings, particularly the number of heads in the MHCA mechanism and the *K* value in the SNF.

**Figure 3:**
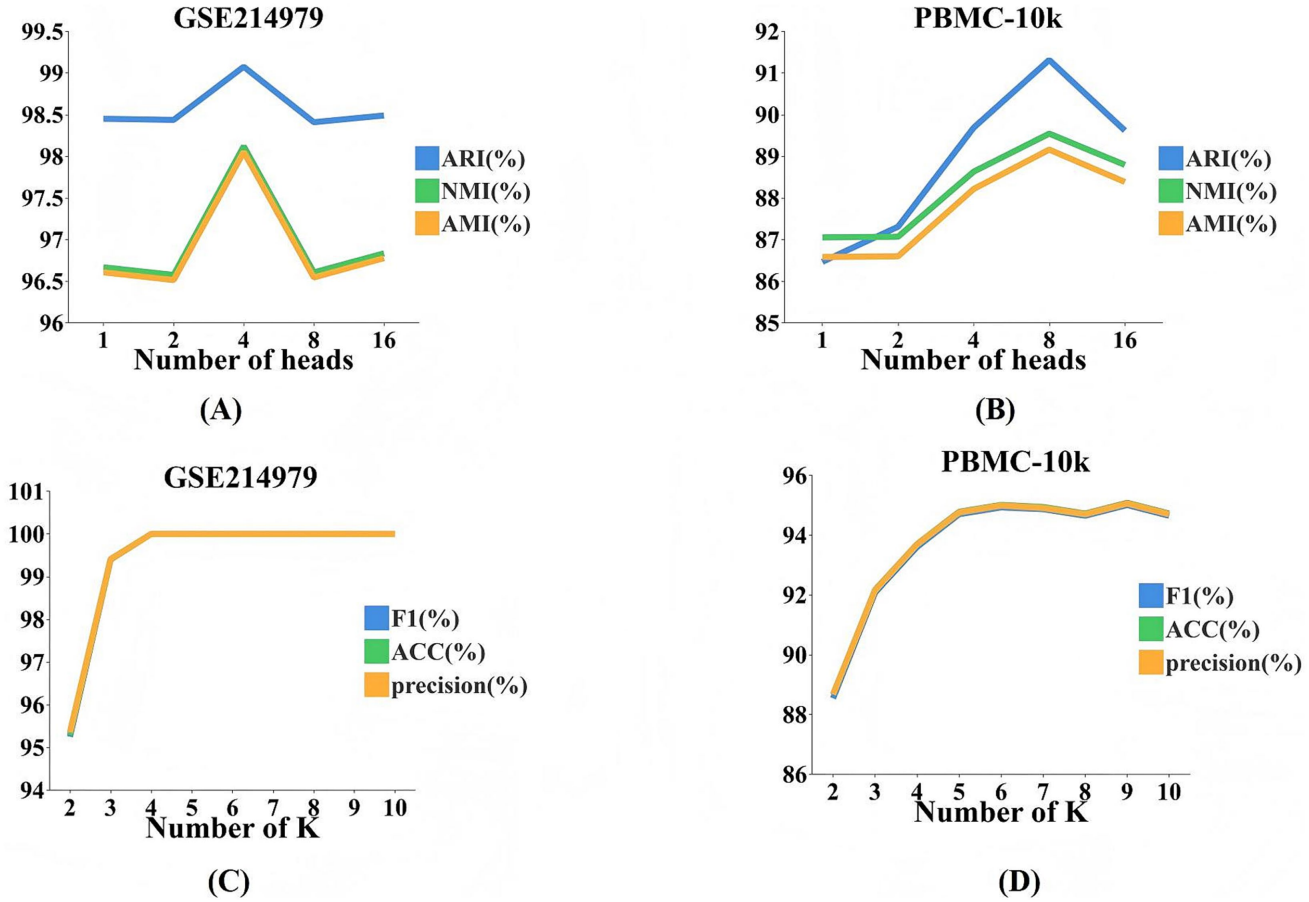
Performance of MOMHCA-SG under various hyperparameter values. **(A)** Results of different heads on the GSE214979 dataset. **(B)** Results of different heads on the PBMC-10k dataset. **(C)** Results of different K on the GSE214979 dataset. **(D)** Results of different K on the PBMC-10k dataset.

As shown in Figure 3A, on the GSE214979 dataset, increasing the number of attention heads from 1 to 4 leads to significant improvements in ARI, NMI, and AMI, indicating enhanced feature interaction. Beyond 4 heads, gains plateau or fluctuate, likely due to redundant information or increased model complexity. A similar trend is observed in Figure 3B for the PBMC-10k dataset, with performance peaking around 4–8 heads, and declining slightly at 16 heads, suggesting possible overfitting or noise amplification.

In classification tasks, tuning the *K* parameter in SNF impacts graph density. In Figure 3C, increasing *K* from 2 to 5 on the GSE214979 dataset boosts accuracy and F1-score, while Figure 3D shows a similar trend for PBMC-10k, with optimal performance around *K* = 6. Further increases yield minimal gains or introduce instability due to over-complex graphs.

### 3.4 MHCA performance analysis

We conducted comprehensive comparative experiments to evaluate the MHCA module within MOMHCA-SG, including feature concatenation, sum/average fusion, self-attention, and multi-view attention mechanisms (Wu et al., 2019). Under identical experimental settings, all methods were assessed for both multi-omics integration and cell type classification tasks. Each method was run 10 times for each of 5 different random seeds (0, 1, 2, 3, 4), and the evaluation metrics were averaged across all runs.

Simple fusion strategies, such as feature concatenation or sum/average, assume that scRNA-seq and scATAC-seq features are fully aligned and equally informative, ignoring modality-specific noise and biological heterogeneity, which results in inferior performance. Self-attention primarily models intra-modality dependencies and is effective for unimodal representation learning but is insufficient for multi-omics integration. Multi-view attention considers weighted contributions from different modalities; however, it typically performs coarse-grained alignment and struggles to handle feature-level sparsity and heterogeneity, thereby limiting its ability to capture fine-grained cross-modal interactions.

In contrast, MHCA treats one modality as queries and the other as key-value pairs, explicitly addressing feature-level misalignment between modalities and enabling fine-grained, token-level cross-modal interactions. The multi-head design allows the model to simultaneously learn multiple complementary alignment subspaces, capturing diverse regulatory relationships, such as promoter accessibility-gene expression coupling and cell-type-specific regulatory programs. By explicitly modeling inter-modality dependencies, MHCA selectively attends to biologically relevant cross-modal features, mitigating the impact of misaligned or noisy signals. This mechanism ensures that the integrated representations reflect true biological correspondences rather than being dominated by modality-specific noise, which is crucial for robust cell type classification.

**Figure 4:**
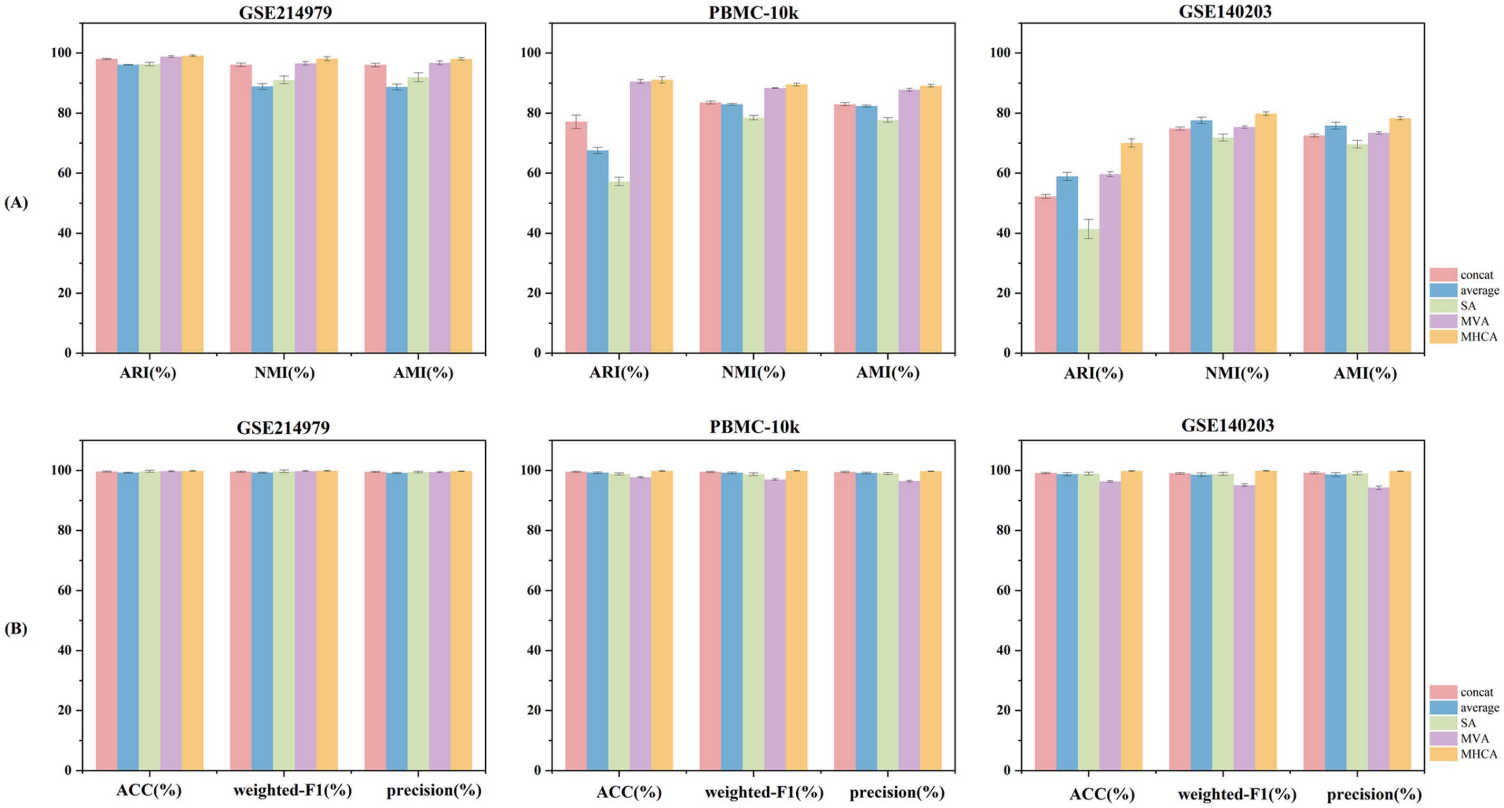
Comparison of feature fusion methods across three datasets. **(A)** Evaluation metrics for scRNA-seq and scATAC-seq integration. **(B)** Metrics for cell type classification.

As shown in Figures 4A,B, MHCA consistently outperforms other attention mechanisms across all metrics for multi-omics integration and cell type classification. These results indicate that, compared to simple fusion strategies and conventional attention mechanisms, MHCA is more effective for cell type classification, and its explicit cross-modal alignment is essential for achieving robust cell type identification.

### 3.5 Bioinformatics analysis

**Figure 5:**
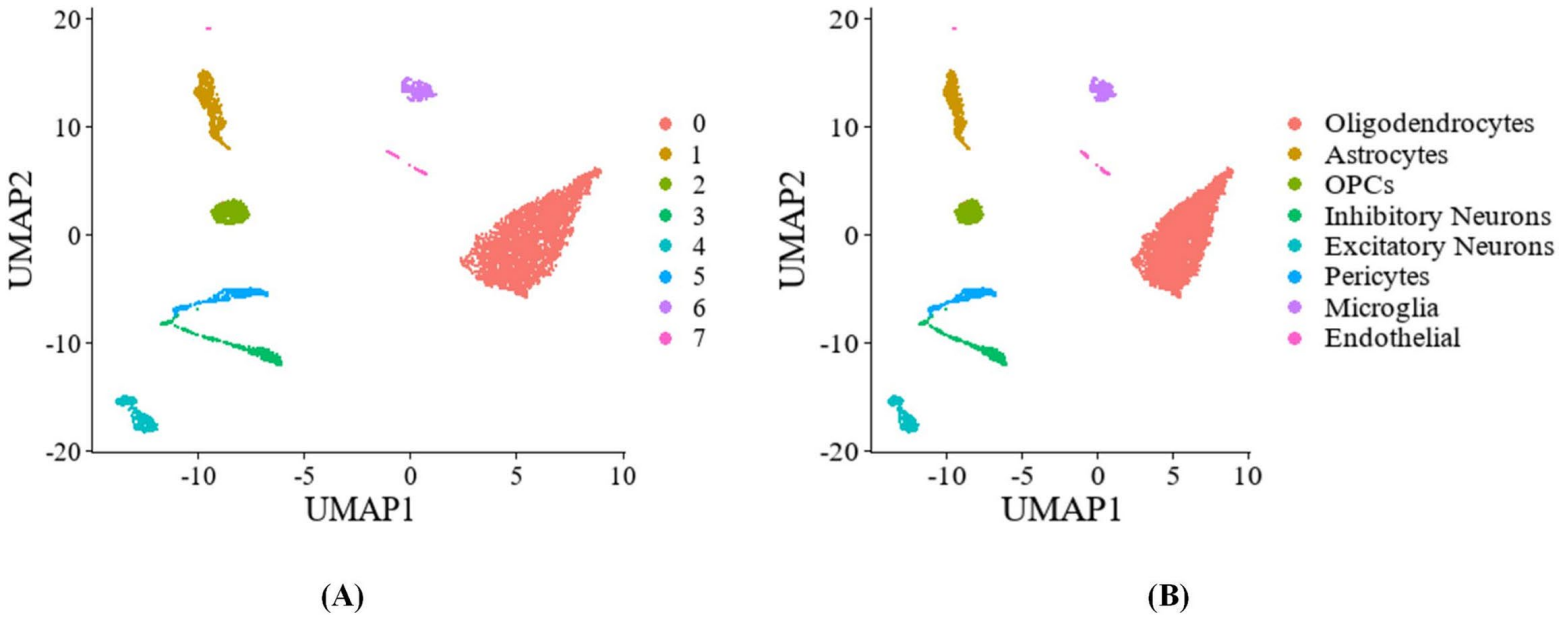
Bioinformatics analysis after MOMHCA-SG classification. **(A)** UMAP plot of the predicted cell type labels after MOMHCA-SG classification on the GSE214979 dataset. **(B)** UMAP plot of the true cell type labels.

This study utilizes Unified Manifold Approximation and Projection (UMAP) for visualization, where the cell type labels predicted by the algorithm are shown in Figure 5A, and the actual cell type labels are depicted in Figure 5B. The results demonstrate that the different cell types are clearly distinguished, and the predicted labels exhibit a high level of consistency with the true labels.

Based on the cell classification results from single-cell multi-omics data, this study conducted differential gene analysis, enrichment analysis, and constructed a protein-protein interaction (PPI) network to gain a deeper understanding of the key molecular mechanisms in AD.

**Figure 6:**
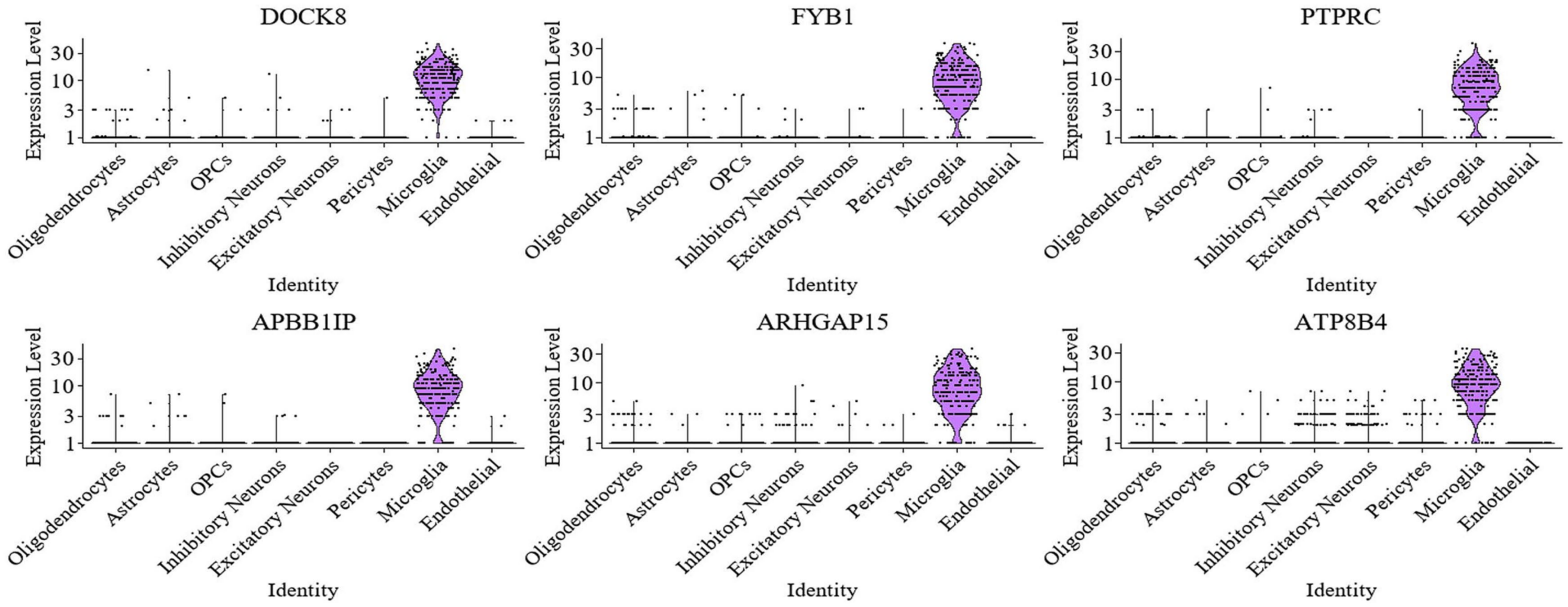
Violin plot of differential gene expression in microglia.

The Seurat package in R was used to identify differentially expressed genes (DEGs) in the preprocessed single-cell multi-omics data from AD. Microglia are crucial immune cells in the brain, and evidence suggests they play a central role in the pathogenesis of Alzheimer’s disease (Hansen et al., 2018), especially in neuroinflammation (Heneka et al., 2015) and the clearance of β-amyloid deposition (Sadigh-Eteghad et al., 2015). In this study, microglia were selected for further analysis. In particular, differential expression analysis was conducted to identify marker genes associated with microglia by comparing the microglia cluster against other cell populations. DEGs between cell populations were identified using Seurat’s FindMarkers and FindAllMarkers functions, which are based on the Wilcoxon rank-sum test. For each comparison, genes were required to be expressed in at least 25% of cells in either group (min.pct = 0.25), with a minimum log2 fold-change threshold of 0.25. *p*-values were adjusted for multiple testing using the Bonferroni correction implemented in Seurat, and genes with adjusted *p*-values below 0.05 were considered statistically significant. Ultimately, genes such as DOCK8, FYB1, PTPRC, APBB1IP, ARHGAP15, and ATP8B4 were found to exhibit significantly differential expression in microglia. These genes may be closely related to immune responses and neuro-inflammation in AD, as shown in Figure 6.

**Figure 7:**
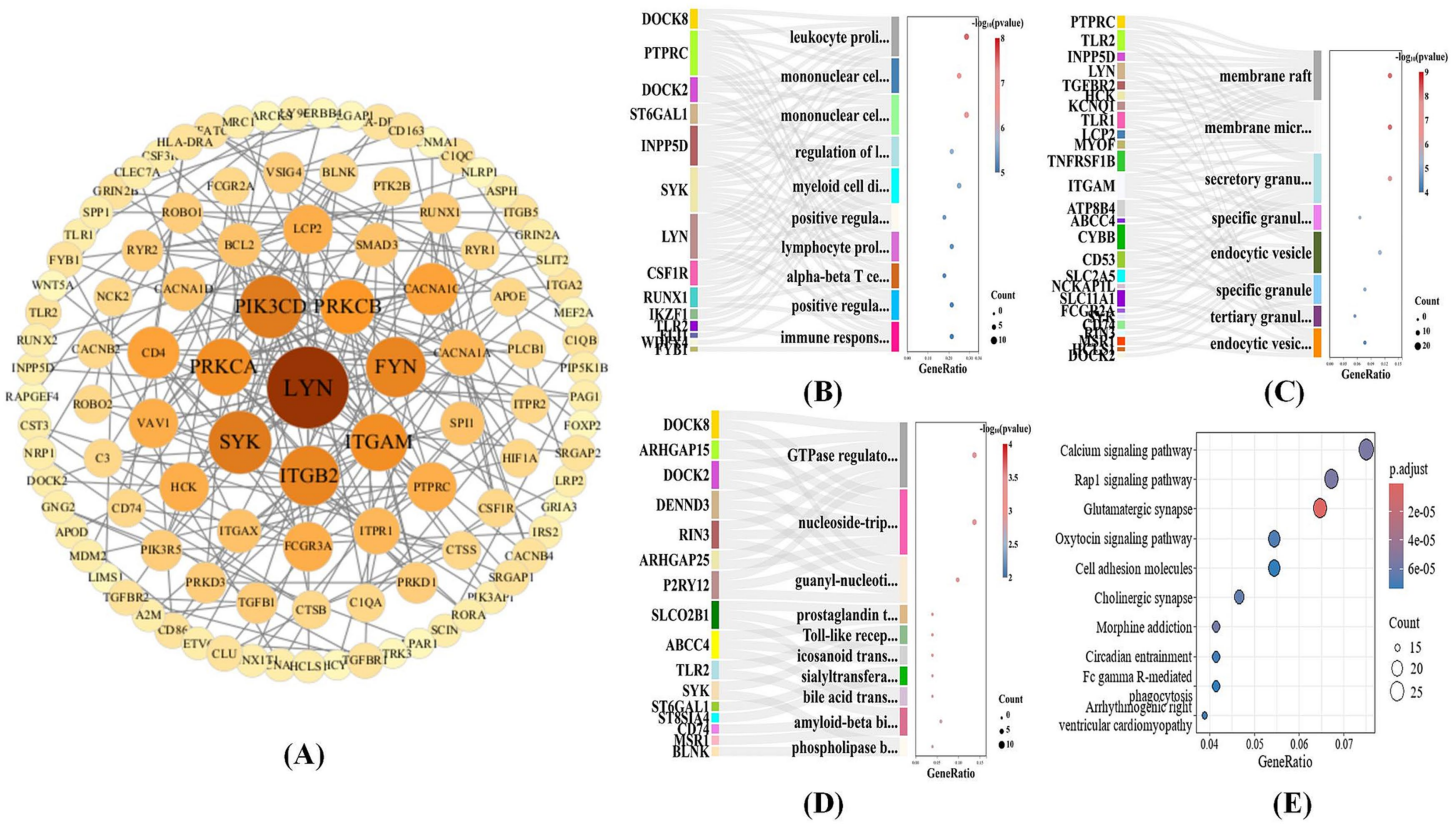
Bioinformatics analysis after MOMHCA-SG classification. **(A)** PPI network of key genes. **(B)** BP enrichment analysis. **(C)** CC enrichment analysis. **(D)** MF enrichment analysis. **(E)** KEGG enrichment analysis of microglia.

All DEGs were submitted to the STRING database for PPI analysis. To gain deeper insights into the key genes within the PPI network, the genes were ranked based on degree centrality. Transcription factors such as LYN, PIK3CD, PRKCB, ITGAM, SYK, FYN, ITGB2, and PTK emerged as top- ranking genes in the network. These transcription factors are crucial in the immune response and neuro-inflammation in microglia, which may be associated with chronic inflammation and neurodegenerative changes in AD, as illustrated in Figure 7A. The degree centrality analysis further demonstrated that the node size of these genes in the PPI network was proportional to their relative importance, underscoring their critical role in the molecular mechanisms underlying Alzheimer’s disease. Gene Ontology (GO) enrichment analysis was performed on microglia, encompassing biological process (BP), cellular component (CC), and molecular function (MF) analyses. Specifically, marker genes identified from the microglia cluster were used as the input gene set for enrichment analysis. GO enrichment analysis was performed using the enrichGO function in the clusterProfiler package, with gene symbols mapped to the human reference genome (org.Hs.eg.db). For each GO category, over-representation analysis was carried out, and statistical significance was assessed using a hypergeometric test. *p*-values were adjusted for multiple testing using the Benjamini-Hochberg (BH) false discovery rate correction, and GO terms with an adjusted *q*-value below 0.05 were considered significantly enriched. As shown in Figure 7B, the results of the BP enrichment analysis revealed that these genes are primarily involved in key biological processes such as immune response, cell proliferation, and differentiation. These processes are closely associated with chronic inflammatory responses in AD, highlighting the crucial role of microglia in immune surveillance and neuro-inflammation in AD. Figure 7C shows the CC enrichment analysis, which indicates that these genes are predominantly enriched in membrane-related structures, such as membrane micro-domains, specific vesicular membranes, and endosomal vesicle membranes. Changes in these membrane structures may be closely linked to microglial activation and amyloid protein clearance functions in AD. Lastly, Figure 7D illustrates the results of the MF enrichment analysis, which suggest that these genes are mainly involved in GTPase regulatory activity, transporter binding, receptor binding (such as Toll-like receptors), and lipid and phospholipid transport. This points to the possibility that microglia regulate neuro-inflammatory responses and intercellular signaling through these mechanisms, potentially exacerbating the pathological progression of AD.

Furthermore, KEGG enrichment analysis was performed on microglia. Marker genes identified from the microglia cluster were first converted from gene symbols to Entrez Gene IDs using the bitr function with the org.Hs.eg.db annotation database. KEGG pathway enrichment analysis was then conducted using the enrich KEGG function with the organism parameter set to “hsa.” Over-representation analysis was performed based on a hypergeometric test. Pathways with a nominal *p*-value <0.05 were considered significantly enriched. *p*-values were adjusted for multiple testing using the BH method as implemented in clusterProfiler. All annotated human genes in the KEGG database were used as the background gene set. As shown in Figure 7E, the pathways that were significantly enriched include the calcium signaling pathway, Rap1 signaling pathway, glutamatergic synapse, and oxytocin signaling pathway. These pathways are crucial for essential biological processes such as cell signal transduction, synaptic transmission, and neuronal regulation. Microglia may mediate these signaling pathways to play a central role in synaptic toxicity, neuro-inflammatory responses, and neurodegenerative changes in AD. These findings underscore that microglia are not only critical in neuronal signal transmission, synaptic function, and intercellular interactions, but may also play a pivotal immune regulatory role in the pathological progression of AD.

### 3.6 Time complexity calculation

The computational complexity of the MOMHCA-SG framework is analyzed in detail. The model comprises four main components: the AE, MHCA fusion, cell similarity network construction, and GCN-based classification.

The AE module processes input features of dimension 𝑑 and reconstructs them into dimension 𝑘, yielding an encoding-decoding complexity of *O* (*Ndk*), where *N* is the sample size. The MHCA module integrates two omic modalities with complexity *O* (*Nh* ( $k_{1}^{2}+k_{2}^{2}+k_{1}k_{2}$ )), where $k_{1}$ and $k_{2}$ are feature dimensions of the two datasets and *h* is the number of attention heads.

In addition, cell-to-cell distance computations contribute *O* (*N*^2^*d*) to the total cost. The SNF-based cell similarity network construction introduces another component with complexity *O* (*N*^2^*m*), where *m* is the number of network layers.

For the GCN classifier, the cost depends on both the adjacency matrix *N* × *N* and the node feature matrix *N* × *d*. With *L* layers, each having *f* hidden units, the total cost is *O* (*LN*^2^*f* + *LNdf*).

Summing all components, the overall time complexity can be approximated as: *O* (*N* ( $d_{1}k_{1}$ *+* $d_{2}k_{2}$ ) *+ Nh* ( $k_{1}^{2}$ *+* $k_{2}^{2}$ *+* $k_{1}k_{2}$ ) *+ N*^2^ (*d + m + Lf*) *+ LNdf*). The dominant term is *O* (*N*^2^), consistent with typical graph-based models.

All experiments were conducted on a high-performance server with a 12-core Intel Xeon Platinum 8,352V CPU@2.10 GHz, 90 GB RAM, and a 32 GB GPU, enabling efficient model training and inference within acceptable runtime. Despite this, MOMHCA-SG demands more memory than conventional deep learning models. Future work should explore strategies to reduce its computational load.

## 4 Discussion

### 4.1 Contribution

This study proposes a unified framework, MOMHCA-SG, for the integration and downstream analysis of single-cell multi-omics data. The model is designed to address three prevalent challenges in multi-omics research: inconsistent dataset distributions, omics heterogeneity, and high dimensionality. MOMHCA-SG integrates AE-based representation learning with MHCA, contrastive learning, SNF-based fusion, and graph convolution, and achieves superior performance on AD-related scRNA-seq and scATAC-seq datasets (GSE214979). In integration tasks, MOMHCA-SG consistently outperforms representative baseline models, including scMCs, SSGATE, and scMDC, demonstrating a clear advantage in extracting and aligning cross-omics cellular relationships. In classification tasks, MOMHCA-SG maintains consistently high performance across three independent datasets, surpassing methods such as MoGCN, MOGAT, and scMoMtF. Collectively, these findings indicate that MOMHCA-SG provides a robust and versatile solution for multi-omics integration and cell-type classification in complex disease settings, with particular applicability to AD research.

The superior empirical performance of MOMHCA-SG can be attributed to its multi-stage representation learning and fusion strategy. First, an AE is employed to extract key features from multi-omics data. Second, MHCA and contrastive learning enable the model to capture shared and complementary signals across omics layers, thereby improving alignment under heterogeneity and helping to mitigate spurious correlations arising from omics-specific noise. Third, SNF provides an adaptive mechanism for integrating similarity structures across omics, while the GCN further refines cell representations by leveraging neighborhood context, enhancing the identification of coherent cellular manifolds. Beyond predictive accuracy, bioinformatics analyses indicate that this framework can prioritize genes and pathways associated with cell identity; notably, the enriched pathways (e.g., the calcium signaling pathway and the Rap1 signaling pathway) are consistent with existing literature, supporting the biological plausibility of the learned features.

### 4.2 Limitations and future directions

Although MOMHCA-SG demonstrates strong empirical performance across multiple tasks, several limitations remain, particularly with respect to real-world deployment in clinical and biomedical settings. First, scalability may become a practical bottleneck. Because the framework integrates multiple components—including AEs, MHCA, contrastive learning, SNF, and GCN—it may incur substantial computational and memory overhead when applied to high-throughput, large-cohort, or high-dimensional single-cell multi-omics studies. Future work will investigate more efficient graph construction and cross-omics fusion mechanisms, such as approximate neighborhood sampling and model compression, to reduce computational complexity while maintaining predictive performance.

Second, data heterogeneity in clinical environments is typically far greater than that observed in carefully curated public datasets. Variations across centers in sample preparation protocols, sequencing platforms, batch effects, and cohort composition can induce distribution shifts that compromise model generalization. Moreover, missing omics modalities are common in practice (e.g., some samples may only have scRNA-seq but lack scATAC-seq). To enhance robustness under real-world conditions, future studies will conduct systematic evaluations in cross-site and cross-platform settings and incorporate adaptive learning strategies and uncertainty estimation to mitigate the impact of distribution shift and incomplete modalities.

Third, translating AI models into healthcare requires careful consideration of regulatory and governance constraints, including interpretability, reproducibility, privacy protection, and fairness/bias assessment. Accordingly, MOMHCA-SG should be positioned as a decision-support tool for clinical and research use rather than an autonomous diagnostic system, and its outputs should be reviewed and interpreted with domain-expert involvement. Future work will integrate explainability modules to provide feature attributions and quantified confidence, establish standardized reporting and external validation protocols, and incorporate expert feedback to calibrate and iteratively refine model outputs.

Overall, future research will focus on improving adaptability, extending the framework to additional omics modalities, reducing computational cost, and strengthening explainability and validation, thereby enhancing the practical utility of MOMHCA-SG in precision medicine and biomedical research.

## 5 Conclusion

This study presents the MOMHCA-SG model, a novel approach for integrating and analyzing single-cell multi-omics data. It is specifically designed to overcome the challenges of data distribution inconsistency, omics heterogeneity, and high dimensionality, which are commonly encountered by existing methods when handling diverse omics datasets. By incorporating dimensionality reduction via AEs, MHCA, contrastive learning, SNF, and GCN, the MOMHCA-SG model significantly improves cell-type classification accuracy and enhances the interpretability of omics features. MOMHCA-SG demonstrated exceptional performance on AD-related scRNA-seq and scATAC-seq datasets (GSE214979).

In integration tasks, the model successfully addresses issues related to data distribution inconsistency and omics heterogeneity by adaptively learning the relationships between different omics data, outperforming methods such as scMCs, SSGATE, and scMDC. This highlights MOMHCA-SG’s capacity to extract and integrate complex cellular relationships from multi-omics data. In classification tasks, MOMHCA-SG significantly outperformed existing methods (e.g., MoGCN, MOGAT, and scMoMtF), with classification accuracy consistently exceeding 95% across additional datasets. These results underscore the model’s robustness and generalizability across diverse datasets and tasks. This study shows that MOMHCA-SG not only overcomes the limitations of traditional algorithms but also provides a novel approach to analyzing multi-omics data in the context of complex diseases, with broad potential applications, particularly in AD research. Bioinformatics analyses reveal that MOMHCA-SG helps identify key genes and pathways driving cell classification. While no entirely new biological mechanisms were identified in this analysis, the pathways detected (such as the calcium signaling and Rap1 signaling pathways) align with existing literature, further supporting the biological relevance of the findings. Future work will focus on enhancing the model’s adaptability, incorporating additional omics data, reducing computational complexity, and integrating explainability analysis, with the goal of advancing MOMHCA-SG’s application in precision medicine and biomedical research.

## Data Availability

The original contributions presented in the study are included in the article/supplementary material, further inquiries can be directed to the corresponding author.

## Footnotes

1
https://www.ncbi.nlm.nih.gov/geo/query/acc.cgi?acc=GSE214979

2
https://www.ncbi.nlm.nih.gov/geo/query/acc.cgi?acc=GSE140203
